# What shapes trust in healthcare? Socio-economic and structural determinants of trust in formal and traditional health providers across 111 countries

**DOI:** 10.1016/j.ssmph.2026.101938

**Published:** 2026-06-23

**Authors:** Christoph Henking

**Affiliations:** Department of Social Policy and Intervention, University of Oxford, Barnett House, 32 -37 Wellington Square, Oxford, OX1 2ER, UK

## Abstract

**Background:**

Public trust in healthcare professionals is crucial for effective healthcare systems. This study examines global variations in trust, assessing how national income and individual socio-economic status relate to trust in formal healthcare professionals and trust in traditional medicine practitioners, and whether institutional and healthcare system characteristics help explain these patterns.

**Methods:**

The study combines cross-sectional data from the Wellcome Global Monitor 2020 (N = 117,088) covering 111 countries with country-level indicators on healthcare expenditure, quality of care, and corruption perception. Multilevel regression models were used to estimate the associations between trust in healthcare professionals (medical doctors and nurses) and traditional healers (both measured on single-item 4-point scales with SD = .8) with a range of individual and country-level predictors.

**Findings:**

Individuals in the highest within-country income quintile reported more trust in health professionals (.04, 95% CI[.03, .06]), but significantly less trust in traditional healers (−.08, [–.09, −.06]) compared to the lowest within-country income quintiles. In middle- and high-income countries there is a strong positive relationship between GDP and trust in healthcare professionals, whereas this association is absent in low-income settings. Similarly, the within-country income gradient with trust in healthcare professionals is absent in low/lower-middle income countries. Finally, lower levels of corruption were positively associated with trust in healthcare professionals (.15, [.07, .23]).

**Interpretation:**

Trust in healthcare professionals is socially stratified, with higher levels among more advantaged groups in wealthier countries, while such gradients are largely absent in lower-income settings. This suggests that socio-economic differences in trust are context-dependent and may emerge only where healthcare systems are sufficiently developed to shape experiences of care. Perceived corruption is associated with trust, highlighting the role of institutional legitimacy. In contrast, trust in traditional practitioners follows an inverse socio-economic pattern, reflecting structural differences in access to care.

## Highlights

• This study reveals global determinants of trust in biomedical and traditional healthcare providers.
• Trust in healthcare professionals is highest among the richest individuals in the wealthiest nations.
• Socio-economic gradients in trust emerge only in middle- and high-income countries.
• Trust in traditional providers is higher among poorer groups within countries.
• Lower perceived corruption is linked to greater trust in healthcare professionals.

## 1 Introduction

Public trust in healthcare professionals is a central element to the functioning of health systems and the effectiveness of health policies (Birkhäuer et al., 2017). But public trust cannot be taken for granted – especially since the COVID-19 pandemic and the political polarisation has strained the public perception of healthcare providers worldwide (Lazarus et al., 2022; Perlis et al., 2024; Van Bavel et al., 2024). Overall, less than half of the world's populations report high levels of trust in health professionals (Moucheraud et al., 2021), and trust tends to be lower in disadvantaged socio-economic groups (Antinyan et al., 2021; Brown et al., 2009). Crucially, low levels in trust are associated with reduced healthcare utilisation, lower treatment satisfaction, and worse health outcomes (Birkhäuer et al., 2017; Douglass & Calnan, 2016; Henking et al., 2023). At the same time, the use and trust in alternative and traditional medicine is widespread globally – especially where formal, biomedical providers are perceived as inaccessible or insufficient (Fakih et al., 2022; World Health Organization, 2019, 2025). Understanding how trust is distributed across different types of healthcare providers has therefore become an important question with direct implications for population health.

Despite increasing attention to this issue several crucial knowledge gaps remain. First, while there is some evidence on socio-economic gradients (Antinyan et al., 2021; Brown et al., 2009; Majumder et al., 2015), it is unclear if economic resources at country or individual level are consistently associated with higher trust. Existing cross-national studies document substantive variation (see Moucheraud et al., 2021), but offer limited theoretical grounding and little insight into the mechanisms linking economic resources to trust, or how these relationships vary across institutional contexts.

Second, the role of trust in traditional and complementary medical providers is underexplored in global comparative research. Across many countries and cultures, traditional and alternative medicine play a central role in healthcare systems, either as substitutes or complements for biomedical care (Puckree et al., 2002; Van der Schee & Groenewegen, 2010; Xu & Yang, 2009). However, little is known about the determinants of trust in these providers, particularly in direct comparison to biomedical professionals.

Third, beyond economic resources, healthcare system and political factors such as quality of care, healthcare investment and corruption may shape trust (Naher et al., 2020; Radin, 2013), but their role has not been systematically examined in a global comparative framework.

This study addresses these three research gaps using a cross-national dataset of 111 countries, and analyses the determinants of trust in biomedical and traditional healthcare providers. Building on relational and institutional perspectives of trust, this study shows how socio-economic gradients are context-dependent, compares trust in biomedical and traditional healthcare providers, and highlights institutional conditions, particularly corruption, as key factors shaping trust across healthcare systems.

### 1.1 Background and research questions

#### 1.1.1 The determinants of trust in healthcare professionals

Trust in healthcare professionals is a multi-dimensional construct shaped by interpersonal relationships and the institutional contexts in which care is delivered. It does not merely reflect an individual attitude, but emerges from experiences within healthcare systems (Gilson, 2003, 2006; Hall et al., 2001).

At the relational level, trust refers to the interpersonal relationship between patients and healthcare professionals, in which patients rely on providers’ expertise and intentions under conditions of uncertainty and vulnerability (Gilson, 2003; Hall et al., 2001).

At the institutional level, trust also depends on whether healthcare systems and its institutions (e.g., hospitals and practices) are perceived as competent, fair and reliable – and whether they fulfil the social contract between institutions and citizens (Bloom, Standing, & Lloyd, 2008; Gilson, 2006). Access to care and the quality of services provided play an important role in these perceptions. In turn, healthcare systems are embedded within wider welfare and political institutions, whose governance and institutional legitimacy also shape trust (Rothstein & Stolle, 2008; Uslaner, 2017).

These relational and institutional dimensions suggest how trust may be patterned by social inequalities. Individuals in different socio-economic positions are exposed to unequal access to care and differences in the quality of healthcare systems, which translate into distinct experiences with providers and healthcare institutions (Mohseni & Lindstrom, 2007; Armstrong et al., 2006).

For example, people with socio-economic advantage are more likely to access high-quality medical treatments, especially in wealthy countries (Fjaer et al., 2017). In contrast, those experiencing financial hardship and members of marginalised societal groups with limited access to quality of care and lower perception of services as competent, fair and legitimate may exhibit lower levels of trust in health professionals (Akafu et al., 2023; Majumder et al., 2015). These patterns suggest that socio-economic differences in trust are created through systematically unequal experiences with healthcare systems and providers.

At the country level, national wealth is expected to increase trust by enabling greater investment in healthcare systems (Baltagi et al., 2017; Watkins et al., 2018). However, evidence suggests that the translation of national income into trust in health is not straightforward and varies between regions (Moucheraud et al., 2021). Further, we do not know if socio-economic gradients in trust vary systematically across countries and under which conditions these socio-economic gradients emerge. Some European evidence suggests that inequalities in trust may be less pronounced in countries with more universal healthcare systems (Wendt et al., 2010), but global comparative evidence remains limited. The first goal of this study is therefore to examine whether national income and individual socio-economic status are consistently associated with trust in healthcare professionals, or whether such associations emerge only under specific country contexts.

Research Question 1 **(individual SES, national income and trust in healthcare** **professionals)**: What is the global relationship between personal SES, national income (GDP) and trust in healthcare professionals?

#### 1.1.2 The determinants of trust in traditional and complementary health practitioners

Traditional and complementary medicine represent important but highly heterogeneous components of healthcare systems worldwide. Its meaning, usage and institutional integration vary substantially between global regions (Fakih et al., 2022; World Health Organization, 2025). In African, Asian and Latin American traditional health providers, they often constitute a primary point of care, particularly in rural areas (Ae-Ngibise et al., 2010; Krah et al., 2018), while in countries such as China, Japan, Korea and India they are more formally integrated into the public health systems (Fakih et al., 2022; Xu & Yang, 2009). In Western countries, non-conventional medicine is typically used as a complementary option besides biomedical care. This often includes natural, herbal, homeopathic or manual therapies (Van der Schee & Groenewegen, 2010; von Schoen-Angerer et al., 2023). These differences suggest that “traditional medicine” does not represent a uniform category, but rather reflects diverse healthcare arrangements shaped by cultural, structural, and political factors (Fakih et al., 2022; World Health Organization, 2025).

Trust in traditional practitioners reflects a combination of relational, and structural factors (Fakih et al., 2022; Gilson, 2006), with several theoretical perspectives offering distinct explanations. Cultural continuity perspectives emphasise the importance of belief systems and shared understandings of illness and healing across different contexts (James et al., 2018). Structural perspectives highlight exclusion from, or barriers to, formal healthcare system. Such exclusion and barriers may lead individuals to rely on more accessible providers, such as traditional practitioners, who are affordable and embedded in communities (Fakih et al. , 2022; Mohseni & Lindstrom, 2007, Puckree et al., 2002). Institutional perspectives suggest that trust in traditional medicine may partly reflect distrust in formal institutions. These accounts are closely linked to postcolonial perspectives, that emphasise historical experiences of enforced biomedical authority and the marginalisation of indigenous knowledge systems (Lowes & Montero, 2021; World Health Organization, 2019).

These perspectives suggest that socio-economic differences in trust in traditional medicine depend on cultural background, access to formal care, institutional conditions, and the organisation of healthcare systems. Some prior evidence suggests that within low- and middle-income countries (LMICs), lower SES individuals tend to use traditional medicine more frequently, while in high-income countries (HICs), alternative forms of medicine might be used by higher SES individuals as a complementary option to biomedical care (World Health Organization, 2019).

However, despite the widespread use of traditional and alternative medicine globally, there is little systematic evidence on how trust in these providers compares to trust in biomedical professionals. (Fakih et al., 2022; World Health Organization, 2019). Trust in traditional medicine practitioners has also received little attention in research on the political economy of healthcare and discussions on trust in healthcare institutions. This study addresses this gap by examining trust in traditional practitioners and contrasting it with trust in biomedical providers, providing new empirical and theoretical insights into how different mechanisms operate across healthcare systems.

**Research question 2 (individual SES, national income and trust in traditional health practitioners)**: What is the global relationship between personal SES, national income (GDP) and trust in traditional medicine practitioners?

#### 1.1.3 Beyond personal SES and GDP: what political and healthcare-specific factors determine trust?

While national income and within-country socioeconomic advantage are expected to be important determinants of trust, they are unlikely to capture the full set of mechanisms shaping trust in healthcare. Building on the conceptualisation of trust from an institutional perspective, this study examines whether healthcare system characteristics and broader political conditions help explain cross-national variation in trust.

From an institutional perspective, trust in healthcare professionals depends on whether healthcare systems are perceived as competent, fair, and reliable (Gilson, 2006; Mackey & Liang, 2012). One key dimension is the performance of healthcare systems, including the service availability and quality. Lower trust in professionals in LMICs may stem from limited availability of services and poorer quality of care (Antinyan et al., 2021; Zhang et al., 2010). More prosperous countries, by contrast, are able to provide universal healthcare to all citizens and invest in long-term care (Baltagi et al., 2017). Similarly, high personal out-of-pocket costs for treatment may undermine trust by raising concerns about access and the motivations of decision-making (Cunningham, 2009; Yuan & Lee, 2022). Where formal biomedical care is inaccessible and connected to higher costs, trust may instead shift toward traditional healers if they are locally more available and socially embedded (World Health Organization, 2025).

Beyond system performance, the broader political context plays a central role. Corruption and weak governance undermine the institutional legitimacy by signaling that public institutions do not operate according to fair and predictable rules (Glynn, 2022; Thompson, 2018). This not only erodes trust in healthcare institutions, but also shapes perceptions of the competence and intentions of healthcare professionals operating within these systems (Gilson, 2006; Mackey & Liang, 2012). Empirically, countries with weaker governance and higher corruption levels tend to have lower levels of trust across institutions and professions (Delhey & Newton, 2003; Uslaner, 2017). Corruption, for instance in the form of informal payments or bribery, has also been linked to reduced trust in both healthcare institutions and professionals working in these settings (Naher et al., 2020; Radin, 2013). Therefore, corruption may represent a key determinant of trust in healthcare professionals.

However, it remains unclear whether these institutional dynamics affect trust in traditional practitioners in the same way, or whether they might be seen as a sought-after alternative where the formal health system is affected by low legitimacy and high levels of corruption. For both biomedical and traditional care, the role of political and healthcare-specific factors (as crucial institutional conditions) in shaping trust have not been systematically examined across providers in a cross-national comparative context. This study therefore addresses this aspect in Research Question 3.

Research Question 3 **(healthcare and political factors)**: Beyond national income, what political and healthcare-specific country characteristics (e.g., health system performance, health expenditure or corruption) are associated with trust in healthcare professionals and traditional medicine practitioners?

Taken together, using an individual dataset with representative data from 111 countries and conceptualising trust as a relational and institutionally embedded construct, this study investigates to what extent national income (GDP) and individual-level socio-economic position shape trust in healthcare professionals (RQ 1), trust in traditional medicine practitioners (RQ 2), and how country-level differences might be driven by healthcare and political system characteristics beyond GDP (RQ 3).

## 2 Methodology

### 2.1 Individual-level sample

The analysis is based on the cross-sectional global survey Wellcome Global Monitor 2020 ; Wellcome, 2020) implemented as part of the Gallup World Poll, which collected nationally representative samples in 113 countries. Data was collected between August 2020 and January 2021 using computer-assisted telephone interviews due to the COVID-19 pandemic. The final dataset resulted in 111 countries (*N* = 117,088) due to missing data in Tajikistan and Venezuela. Details on the participating countries and descriptive statistics per country can be found in Tables S1 and S2 in the appendix. The data collection is situated during the COVID-19 pandemic which might have impacted levels of trust in health professionals. We ran sensitivity analyses for the main multilevel regression model in the appendix (Table S3), where we include Covid-19-specific indicators such as lockdown intensity and Covid-19 mortality rates. This analysis shows that my results remain stable when including these factors.

### 2.2 Variables

#### 2.2.1 Outcome variable

Trust in health professionals was measured using the item “How much do you trust the following? Doctors and nurses in this country” on a scale from 1 = not at all, 2 = not much, 3 = some, 4 = a lot. Trust in traditional health practitioners was measured on the same scale from 1 to 4, using the item “How much do you trust the following? Trust in traditional healers in this country”. Notably the survey item may be interpreted differently across cultural and regional contexts. In LMICs, the term typically refers to herbal, spiritual, or community-based healers, while in HICs, it may be understood more broadly to include complementary or alternative medicine providers such as homeopaths or naturopaths (Fakih et al., 2022; Van der Schee & Groenewegen, 2010; World Health Organization, 2019). For the main analysis, the variable was treated as numerical to use the full variance of this item. To validate whether the results are sensitive to a different modelling choice, we also coded binary variables for the trust variables where one category was comprised of the “not at all” or “not much” (coded as 0) and another category as “some” or “a lot) (coded as 1). Based on this alternative coding, we ran logistic regression models in correspondence with the models shown in the main analysis of this paper (see Table 2). The results are shown in the appendix (Table S4) and indicate that the findings remain consistent with the main analysis shown in Table 2.

#### 2.2.2 Individual-level factors

Socio-economic status was measured through education (Primary school or less; completed secondary school; completed tertiary education) and household income (Per Capita Income Quintiles within countries). The study includes “trust in neighbours” as indictor for general societal trust outside the health sector (measured from 1 = not at all to 4 = a lot). Gender and age are also included as control variables because both may influence trust and healthcare-seeking behaviour.

#### 2.2.3 Country-level factors

National income was measured using gross domestic product per capita (GDP) and it was downloaded from the World Bank (2022) open data website alongside health expenditure as percentage and out-of-pocket health expenditure (both as percentage of GDP). The Healthcare Access and Quality (HAQ) index, created by the Institute for Health Metrics and Evaluation (IHME) at the University of Washington was used to measure quality of care (Haakenstad et al., 2022). It was downloaded for the last available year of 2015 from the EU Joint Research Center website (EU Joint Research Centre, 2025). Finally, we used the Transparency International Corruption Perception Index for the year 2020 which uses a scale from 1 to 100 to measure the level of perceived corruption per country. Higher values indicate lower levels of corruption in each country (Transparency International, 2021). All country level values for these indicators – including all countries’ income categories – are shown in the appendix (Table S2).

#### 2.2.4 Statistical analysis

To test the three research questions, we ran a multilevel regression analysis where individuals are nested in countries and the main outcome variables are (a) trust in health professionals and (b) trust in traditional healers. We first established a random intercept model, and then added predictors according to the variables shown in Table 2. To increase comparability between vastly different scales, several country-level predictors (health expenditure, out of pocket payments, and corruption and quality of care indices) were standardised so that a change of “1” equals to 1 SD.

We ran eight separate models for the two outcomes “trust in health professionals” (M1 - M4) and “trust in traditional healers” (M5 - M8). Models M1 – M3 investigate research question 1, the relationship between national income (GDP), personal SES and trust in health professionals. We contrast household income quintiles 1,3 and 5 as main indicators for within-country socio-economic gradients. Model M1 only uses simple, linear predictors in the model. Models 2 and M3 then add an interaction term and a quadratic term for the relationship on household income and GDP respectively. This is done to test whether the personal income gradient differs by country prosperity and if the relationship between GDP and trust might not be linear but quadratic. The models M4 - M7 are used to examine research question 2, the relationship between national income (GDP), personal SES and trust in traditional healers. The models follow the same construction as M1 - M3.

Models M4 and M8 are used to examine research question 3, the political and healthcare related country determinants of trust. This is done by adding the country-level predictors of health expenditure, out-of-pocket health spending, quality of care and the corruption index to my model for both types of trust. “Trust in neighbours” is added as control variable to control for general trust in other life domains. Gender and age are also used as control variables throughout all models to isolate the effect of demographic factors on trust.

## 3 Results

### 3.1 Descriptive statistics and correlations

**Fig. 1:**
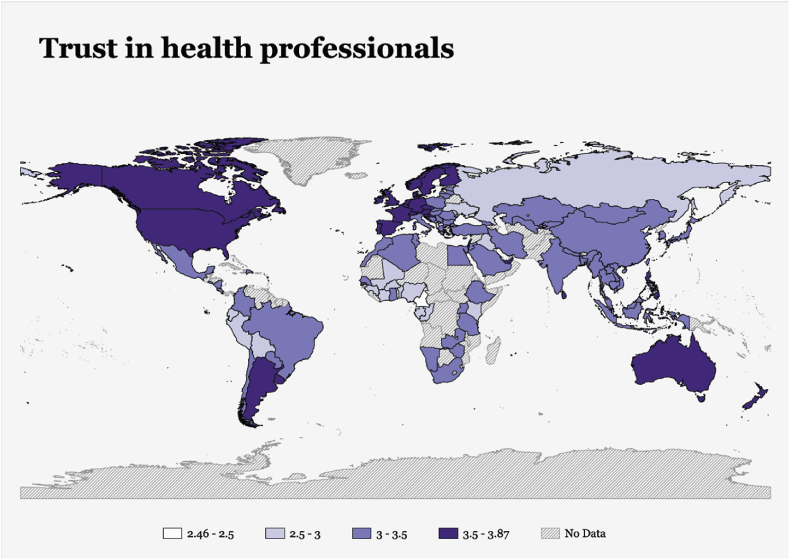
Mean trust in health professionals by country, ranging from scale from 1 = not at all to 4 = a lot ((M = 3.3, SD = .8).

**Fig. 2:**
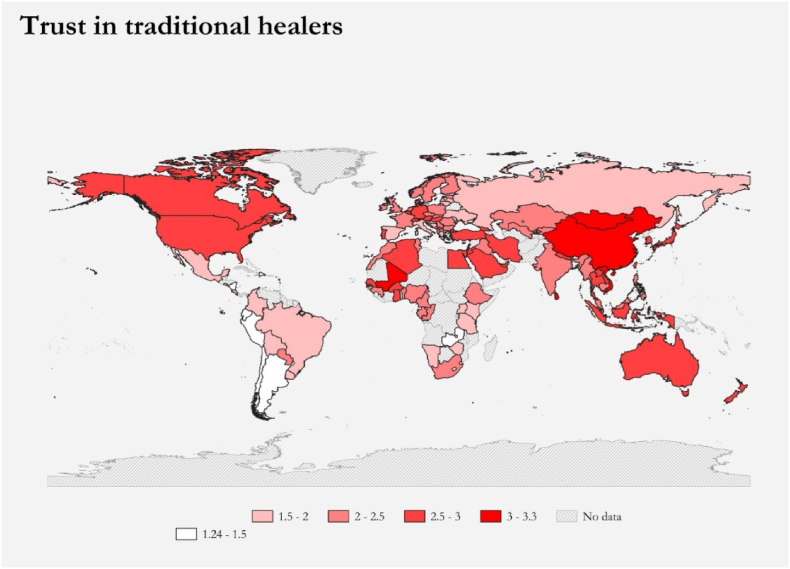
Mean trust in traditional health practitioners by country, ranging from scale from 1 = not at all to 4 = a lot (M = 2.3, SD = 1.0).

Levels of trust in health professionals and traditional health practitioners globally are shown as maps in Fig. 1, Fig. 2 (darker colour indicates higher levels of trust). Fig. 1 reveals regional patterns, where most Western European countries as well as North America display high levels of trust in health professionals. Additionally, descriptive statistics per variables, across all 111 countries, are shown in Table 1. Notably, trust in healthcare professionals is considerably higher (M = 3.3, SD = .8) than trust in traditional healers (M = 2.3, SD = 1.0) across the whole sample of 111 countries.

**Table 1:** Descriptive statistics.

		All countries	High income countries	Low- and middle-income countries
Socio-demographic characteristics	Countries, N	111, N = 117,088	43, N = 43,201	68, N = 73,887
	Gender	F = 50.9%, M = 49.1%	F = 50.7%, M = 49.3%	F = 48.1%, M = 51.9%
	Age (mean, sd)	40.2 years (17.3)	49.1 years (18.1)	35.0 years (12.8)
Country-level indicators	GDP per capita in USD (mean, SD)	15,862 (18,908)	35,340 (18,750)	4,472 (3,076)
	GDP (log) (mean, sd)	8.9 (1.3)	10.3 (.5)	8.1 (.8)
	Healthcare Access and Quality (HAQ) index	68.6 (15.3)	83.6 (5.9)	60 (12)
	Corruption index (mean, sd). Note: higher scores indicate lower corruption	46.6 (17.9)	65.9 (13.2)	35.5 (8)
	Health expenditure as % of GDP (mean, SD)	6.6 (2.6)	8.5 (2.3)	4.6 (1.6)
	% Out of pocket healthcare expenditure	32.3 (15.5)	21.2 (8.9)	44.9 (15.2)
Education (individual level)	Elementary or less	13.3%	7.5%	16.7%
	Secondary	55.1%	53.2%	56.3%
	Tertiary	31.5%	39.3%	27.0%
	Number of responses	116,352	42,980	73,372
Trust in health practitioners (doctors and nurses)	mean (SD)	3.3 (.8)	3.5 (.7)	3.1 (.8)
Trust in traditional healers	mean (SD)	2.3 (1)	2.3 (1)	2.2 (1.1)
Trust in clinics	mean (SD)	3 (.9)	3.3 (.8)	2.8 (.9)
Trust in neighbours	mean (SD)	3 (.9)	3.2 (.8)	2.8 (.9)

**Table 2:** Multi-level regression model.

*Individual-level factors*	Outcome: Trust in health professionals				Outcome: Trust in traditional healers			
	M1 (individual level)	M2 (income ∗GDP)	M3 (quadratic model)	M4 (country-level)	M5 (individual level)	M6 (Income∗ GDP)	M7 (quadratic model)	M8 (country-level)
Age	**.02∗** [.01, .02]	x	x	x	**−.05∗** [–.06, –.05]	x	x	x
Gender (being male)	**−.02∗∗** [–.03, −.01]	x	x	x	**−.03∗∗** [–.04,−.02]	x	x	x
Primary school or less (ref = secondary school)	−.01 [–.02, –.00]	x	x	x	**.07∗∗** [.06, .09]	x	x	x
University education (ref = secondary school)	.**04∗∗** [.03, .05]	x	x	x	**−.08∗∗** [–.10, −.06]	x	x	x
Household income quintile 3 (ref = quintile 1)	**.03∗∗** [.02, .04]	**−.20∗∗** [–.30, −.10]	**.04∗∗** [.02, .05]	x	**−.04∗∗** [–.06, −.02]	−.08 [–.21, .05]	**−.03∗∗** [–.05, −.01]	x
Household income quintile 5 (ref = quintile 1)	**.04∗∗** [.03, .06]	**−.31∗∗** [–.41, −.21]	**.05∗∗** [.03, .06]	x	**−.08∗** [–.09, –.06]	−.01 [–.14, .11]	**−.07∗∗** [–.09, −.05]	x
Quintile 3 ∗ Log(GDP)		**.03∗∗** [.02, .04]				−.01 [–.01, .02]		
Quintile 5 ∗ Log(GDP)		**.04∗∗** [.03, .05]				−.01 [–.02, .01]		
Trust in neighbours	**.20∗∗** [.19, .20]			x	**.14∗∗** [.13, .14]			x
***Country-level factors***								
Log(GDP)		**.11∗∗** [.08, .15]	**−1.01∗∗** [–1.52,−.49]	−.02 [–.08, .14]		.06 [–.01,.13]	**−1.12∗** [–2.11, −.12]	−.01 [–.21, .20]
Log(GDP)^2			**.06∗∗** [.03, .09]				**.07∗** [.01, .12]	
Corruption index				**.15∗∗** [.07, .23]				.15 [–.02,.32]
Health expenditure as % of GDP				.01 [–.05, .07]				**−.14∗** [–.27, −.01]
% Out of Pocket healthcare expenditure				−.02 [–.08, .03]				.05 [–.06,.17]
Quality of care index				.03 [–.08, .14]				.10 [–.11,.30]

Marginal R^2^	.053	.057	.069	.121	.018	.006	.015	.033
Conditional R^2^	.167	.152	.150	.190	.207	.201	.202	.214

***Notes***. "x” indicates that the variable was included as control variable in the model; 111 countries included. Several country-level factors (corruption index, healthcare expenditure, out of pocket expenditure and quality of care) were standardised so that a change of 1 level = 1 SD. ; ∗∗p < 0.01, ∗p < 0.05.

Further, we calculated the mean values of trust for each individual country as shown in the appendix (Table S1). Additionally, we calculated the within-country correlation coefficients between different types of trust to provide a descriptive overview of the variables in the study. As shown in Table S1, Belgium has the highest level of trust in healthcare professionals (M = 3.87), whereas Cameroon has the lowest trust (M = 2.48). In all 111 countries, trust in health professionals (as a person) is higher than trust in hospitals (as a health institution). Within all countries, there is a positive correlation between trust in health professionals and trust in hospitals, ranging from r = .11 in Spain to r = .69 in Russia. Trust in traditional healers is highest in China (M = 3.30) and lowest in Argentina (M = 1.42). Mean trust in healthcare professionals is higher than trust in traditional healers in all countries, with the exception of China and Mali. The overall correlation between trust in health professionals and trust in healers is modest (r = .11). The correlation coefficient is highest in China (r = .42) and the lowest in Spain (r = −.11). This suggests that these two types of trust only have a low correlation and there are substantive differences between countries.

### 3.2 Multilevel-analysis regression analyses

Research question 1 (relationship between individual SES, national income and trust in healthcare professionals)

**Fig. 3:**
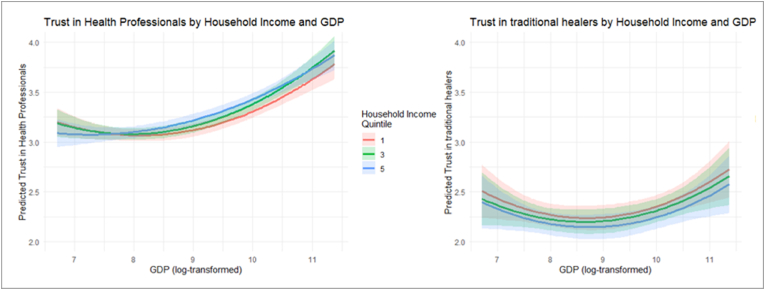
Predicted values of trust in health professionals and traditional healers as a function of GDP and within country income quintile (95% confidence intervals around predictions).

**Fig. 4:**
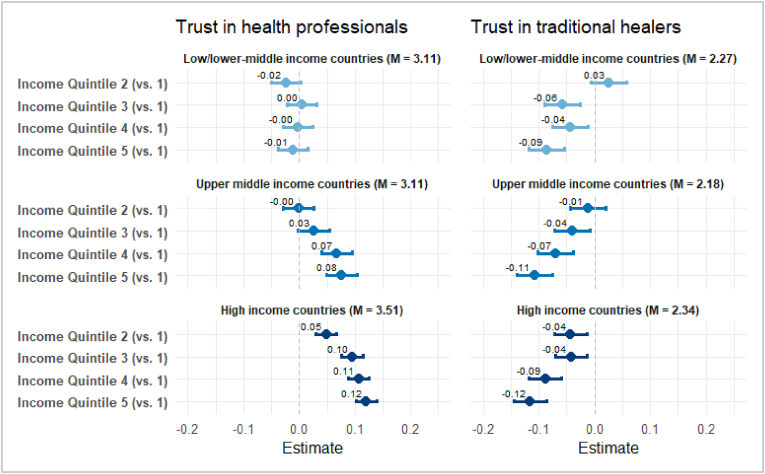
Predicted values of within country household income quintiles, country groups and trust (95% CIs around each predicted value). Income Quintile 1 is used as reference category.

Models M1, M2 and M3 are used to investigate research question 1 and examine the association between personal SES, country-level GDP and trust in health professionals. Model M1 shows that higher household income is linked to higher levels of trust in health professionals across the 111 countries (quintile 3: .033, 95% CI:[.02, .04]; quintile 5: .04, CI[.03, .06]). Model M2 shows that there is a significant interaction between individual-level household income and GDP (quintile 3 ∗ log(GDP) = .03 CI:[.02, .04]; quintile 5 ∗ log(GDP) = .04,CI:[.03, .05]). This suggests that the income-trust gradient becomes steeper in richer countries. Model M3 shows that the quadratic term for GDP is also statistically significant (log(GDP)^2: .06, 95% CI:[.03, .09]), indicating that the association between GDP and trust has a quadratic shape. This finding is further illustrated and visualised in the section below (see Fig. 3) where a steep incline of trust is shown as GDP rises. Overall, the effect sizes and visualisation also suggest that GDP matters more to explain trust compared to personal, within-country income. Fig. 3, Fig. 4 below help illustrate these findings in more detail.

Research question 2 (relationship between personal SES, national income and trust in traditional healers)

Models M5, M6 and M7 are used to investigate research question 2, the association between SES, GDP and trust in traditional healers. Contrary to M1 (for healthcare professionals), M5 shows that being in a higher within country income quintile (quintile 3: −.04, 95% CI:[−.06, −.02]; quintile 5: −.08, 95% CI:[−.09, −.06]) is associated with lower trust in traditional healers. Further, model M6 shows no direct relationship between GDP and trust in traditional health practitioners, and no interaction between household income and trust. This suggest a weak relationship between GDP and trust in traditional health practitioners. However, similar to trust in health professionals, M7 shows that the quadratic term for GDP and trust in traditional health practitioners is significant (log(GDP)^2: .07, 95% CI:[.01, .12]). Fig. 3, Fig. 4 – as described further below – will help illustrate these associations in more detail to help make sense of the findings derived from model M6 and M7.

Research question 3 (healthcare and political factors)

Finally, models M4 and M8 focus on the role of broader country-level factors for both types of trust. Model M4 shows that lower corruption perception (higher corruption index: .15, 95% CI:[.07, .23]) is significantly associated with greater trust in health professionals. Other health system characteristics, including health expenditure, out-of-pocket expenditure, and quality of care, are not significantly associated with trust in health professionals. Model M8 shows that only health expenditure is significantly and negatively associated with trust in traditional healers (−.14, CI:[−.27, −.01]), meaning that an increase of 1 SD in health expenditure is related to a .14 decrease on the 4-point-scale in trust in traditional health practitioners. Notably, in both model M4 and M8, the associations between GDP and trust become non-significant. This suggests that the addition of healthcare and political predictors interferes with this relationship and, especially corruption (for healthcare professionals) and health expenditure (for traditional healers), might mediate the relationship of GDP and trust outcomes. While causal mediation is difficult to test in cross-sectional data (Schuler et al., 2025), future longitudinal research should investigate these pathways.

### 3.3 Visualising the relationship of individual income, GDP and trust

The multilevel regression model (Table 2) revealed that both interaction and quadratic terms are substantial for understanding the relationship between individual income, GDP, and both types of trust. To make these relationships more transparent, we used the underlying regression models to plot the predicted values of trust in health professionals and traditional healers as a function of GDP and income, and included both quadratic and interaction terms (see Fig. 3). Additionally, we plotted the income gradients of trust separately for low/middle-, upper-middle, and high-income country groups to illustrate the magnitude and direction of socio-economic gradients (see Fig. 4).

For trust in healthcare professionals (left panel of Fig. 3), the relationship with GDP appears to be relatively flat across lower levels of GDP but then rises more sharply for richer countries. Fig. 4 shows that the income gradient (higher-income households have higher trust in health professionals) becomes apparent only in medium- and especially in high-income countries but is absent in low/lower-middle income countries. In combination, this suggests that only when countries reach a certain threshold of economic development does trust in health professionals start to rise, and within-country socio-economic gradients begin to emerge and widen.

For trust in traditional healers (right panel of Fig. 3), the association with GDP is much flatter compared to the graph on the left. The relationship follows a U-shaped pattern: trust initially declines as GDP rises from lower levels (with a low point at the medium GDP level) but begins to increase again once GDP reaches higher levels. The individual-level income quintiles (as shown in Fig. 4) consistently show that people with lower incomes within countries have higher levels of trust in traditional healers.

## 4 Discussion

Understanding the global variation of trust in healthcare professionals – both biomedical and traditional healthcare providers – is essential for functioning healthcare systems as trust influences treatment utilisation, adherence and health outcomes (Birkhäuer et al., 2017; Douglass & Calnan, 2016; Henking et al., 2023). Building on relational and institutional perspectives of trust, this study investigated how socio-economic position, national income, and institutional conditions jointly shape trust in healthcare professionals and traditional medicine practitioners across 111 countries.

The study finds that trust in healthcare professionals is generally higher than trust in traditional medicine practitioners across countries, but this difference reflects fundamentally different underlying mechanisms shaping trust in the two types of providers. We show that national income is positively associated with trust in healthcare professionals, but only when a certain threshold of economic development is reached (i.e., upper-middle income countries). Similarly, in terms of personal income, socio-economic gradients in trust in healthcare professionals are not universal and only emerge in higher middle- and high-income (and not in lowered income) settings. Crucially, this indicates that these gradients are conditional on economic and institutional contexts. In contrast, for trust in traditional health practitioners, the economic gradient is reversed: people with lower personal income have higher trust and this is consistent across all, suggesting that trust is shaped by structural access constraints and substitution dynamics rather than purely cultural factors.

In addition, the study finds that lower levels of perceived corruption are strongly associated with higher levels of trust in healthcare professionals, This pattern suggests that institutional legitimacy may play an important role in shaping trust in biomedical providers. These key findings are discussed in detail below.

### 4.1 Trust in healthcare professionals

The findings suggest that socio-economic gradients are context dependent as they emerge through different experiences in the healthcare system. That is, individuals living in richer countries and higher socioeconomic status within those countries are likely to better access to high quality services, which can strengthen the perception of competence and fairness in interactions with healthcare professionals. (Akafu et al., 2023; Delhey & Newton, 2003; McAndrew, 2020).

However, while there is a personal income gradient in trust in middle- and high-income countries, there is no personal income gradient in low/lower-middle income countries. That is, where access and quality of healthcare are limited for large parts of the population in lower income settings, these differences are reduced. Thus, it may be the case that in contexts where most individuals are exposed to constrained conditions of care, there is not a direct translation of socioeconomic position into gradients in trust (Mohseni & Lindstrom, 2007; Gilson, 2006). Relatedly, from an institutional perspective, when institutions are perceived as unreliable, under-resourced, or governed by inconsistent rules throughout society, trust in health providers may be uniformly reduced across social groups (Gilson, 2006; Rothstein & Stolle, 2008; Uslaner, 2017).Further, many of the lowest income countries in our sample (e.g., Ethiopia, Mali, Uganda, Congo Brazzaville) have legacies of colonial medical campaigns, which have lowered overall trust in healthcare providers across generations for the entire population rather than specific socio-economic groups (Lowes & Montero, 2021).

Overall, these patterns indicate that socio-economic gradients in trust are conditional on both system performance and institutional legitimacy, rather than universal. The observed differences in trust levels are likely to manifest in social inequalities in healthcare utilisation and treatment uptake (Birkhäuer et al., 2017; Douglass & Calnan, 2016; Henking et al., 2023). Trust may also interact with related psychosocial mechanisms such as societal stigma (as both have shared social determinants), which can further shape healthcare-seeking behaviour, particularly in mental healthcare (Bracke et al., 2019; Henking et al., 2023). The specific link between these two concepts and their consequences for health outcomes should be investigated further.

### 4.2 The role of institutional legitimacy and corruption

The study also shows that there is a strong relationship between perceived corruption and trust in healthcare professionals – which highlights the importance of institutional legitimacy of public institutions. Notably, this study shows that corruption levels, rather than healthcare-specific factors such as health expenditure and healthcare quality measures (Baltagi et al., 2017; Cunningham, 2009; Zhang et al., 2010), are associated with trust in health providers globally.

Corruption can be understood as a direct and visible signal of institutional unfairness. Where corruption is perceived as high, this erodes the perception of institutional fairness and legitimacy throughout society and public organisations (Delhey & Newton, 2003; Lavallée et al., 2008; Uslaner, 2017). Thus, this mistrust carries over to the trust of healthcare providers (Gilson, 2006). Corruption and institutional unfairness can also be experienced directly in the healthcare sector through bribery, informal payments and unequal treatment from healthcare providers (Naher et al., 2020; Radin, 2013) as well as practices like overpaying, mismanagement of cases and resources, which erodes relationships with healthcare providers both in terms of perceived competence and intentions (Holmberg & Rothstein, 2011; Lewis, 2006).

By contrast, healthcare expenditure and indicators of system performance are more abstract and less directly observable to individuals, especially when captured across countries. They might not necessarily translate into perceived competence or fairness in patient-provider interactions, beyond what is already explained by national income and corruption levels. Trust, as a relational construct, is particularly sensitive to these perceptions (Gilson, 2003; Hall et al., 2001), which helps explain why corruption shows a stronger and more consistent association with trust than expenditure or quality indicators. In doing so, this study moves beyond prior cross-national work, which has primarily documented descriptive variation in trust (e.g., Moucheraud et al., 2021), by identifying institutional legitimacy as a key mechanism linking governance conditions to trust in healthcare professionals.

However, it needs to be noted that healthcare system and overall corruption perception are not clear cut but interact. For example, healthcare system structure, including levels of coverage and funding arrangements, may influence how corruption is perceived. The specific relationship between economic development, corruption and trust should be addressed in future research. One additional, mechanistic hypothesis derived from this research is that corruption might be a mediator in the relationship between GDP and trust. However, causal mediation is difficult to test in cross-sectional data (Schuler et al., 2025), and requires future longitudinal studies to investigate these pathways.

### 4.3 Trust in traditional medicine practitioners

This study provides new evidence on the determinants of trust in traditional practitioners in a global comparative context.

In contrast to biomedical providers, trust in traditional practitioners follows a different pattern, with a relatively weak association with national income and a globally consistent inverse socio-economic gradient within countries. These findings suggest that trust in traditional practitioners is shaped by distinct mechanisms. Trust in traditional practitioners may be more closely linked to structural access constraints and community-embedded forms of care. That is, for lower socio-economic groups within countries, traditional practitioners may represent accessible, affordable, and locally embedded alternatives when formal healthcare systems are limited or exclusionary (Fakih et al., 2022; Puckree et al., 2002; Mohseni & Lindstrom, 2007). In contrast, higher socio-economic groups, particularly in more developed systems, are more likely to rely on biomedical providers, reducing their reliance on and trust in traditional practitioners (World Health Organization, 2019). The finding also suggests that trust in traditional health practitioners extends beyond cultural believe systems (James et al., 2018), and may also reflect socio-economic inequalities in access to biomedical care.

The observed U-shaped relationship between GDP and trust in traditional health practitioners, may reflect differences in healthcare system structure across country contexts. Low and lower-middle income settings, particularly in sub-Saharan Africa, tend to have a stronger reliance on traditional medicine (Krah et al., 2018; Puckree et al., 2002; World Health Organization, 2019) due to the unaffordability of conventional biomedical care (Ae-Ngibise et al., 2010; World Health Organization, 2019). This could explain why trust is generally higher in low-income countries than in middle-income ones, where the healthcare landscape is more mixed.

Further, the study shows that higher healthcare expenditure is associated with lower trust in traditional healers. This evidence may support the idea that investments in formal healthcare systems may reduce reliance on traditional healing practices.

Finally, It is also important to consider that the interpretation of the concept of “traditional healer” may differ across countries, which may affect the comparability of responses. In high-income countries, nonconventional medicine is most typically referred to as supplementary and alternative medicine rather than traditional healing practices. As such, these forms of care were likely seen more as supplementary care, rather than a substitute for conventional care. Future research should capture such definitional differences more clearly.

### 4.4 Limitations

This study has several important limitations. First, there may be differences in the interpretation of “trust in traditional healers,” which likely captures something different from the use of alternative medicine in high-income countries. Relatedly, the items used in this research underwent cognitive testing in different countries, uncertainty over the interpretation of questions and concepts across different cultures remain an important limitation.

Second, outcome variables are measured using a single items on trust on a four point scale which limits data quality and warrants caution in the interpretation of findings. Third, the cross-sectional nature of this study hinders the causal interpretation of the relationships between the variables in this study. Fourth, it should be noted that the country sample is somewhat limited. In particular, only five low-income countries are included all situated Sub-Saharan Africa (Burkina Faso, Ethiopia, Guinea, Mali, Uganda). However, in the main analyses of socioeconomic gradients (see Fig. 4) these countries were grouped with lower- and middle-income countries from Africa, Asia, and South America, increasing this analytical group to 28 out of 111 countries to help mitigate this limitation.

Finally, the data collection during COVID-19 may have influenced levels of trust and healthcare perceptions. To account for this, supplementary analyses including COVID-19-related indicators suggest that the main findings are robust and the survey items are designed to capture general attitudes beyond the specific pandemic context. Nonetheless, future research should examine whether the observed dynamics and social gradients remain stable.

Future research should use longitudinal designs, more thorough measurement instruments and link variations in trust to health outcomes. Causal research should also further examine the role of corruption and healthcare system characteristics in shaping trust across different provider types, for example, using mediation analyses.

## 5 Conclusion

This paper reveals global patterns and determinants of trust in both biomedical and traditional healthcare providers. Trust in biomedical providers is socially stratified, with higher levels among the richest individuals in the wealthiest countries, however, these socio-economic gradients are largely absent in low/lower-middle income countries. This indicates that socio-economic differences in trust are context-dependent and emerge primarily where healthcare systems are sufficiently developed to translate socio-economic position into differentiated experiences of care. Beyond national income, perceived corruption levels were identified as an important correlate of trust in healthcare professionals, highlighting the role of institutional legitimacy.

In contrast, trust in traditional medicine practitioners follows an inverse socio-economic pattern suggesting that it reflects structural differences in access to care beyond cultural preferences. Overall, trust in healthcare is shaped not only by socio-economic position or national wealth, but by institutional and structural conditions that influence access to and experiences of care, with important implications for health inequalities. In times of rising political polarisation and growing public distrust in science, these patterns need to be recognised and monitored closely.

## Ethical statement

This study uses publicly available, anonymised secondary data and did not require ethical approval.

## Funding

This work was supported by the Economic and Social Research Council (ESRC) [grant number ES/P000649/1].

## Declaration of interest

The author declares no conflict of interests.

## Data Availability

Data will be made available on request.
