# Supplementary material for: What shapes trust in healthcare? Socio-economic and structural determinants of trust in formal and traditional health providers across 111 countries
Source: SSM Popul Health. 2026 Jun 23;35:101938. doi: 10.1016/j.ssmph.2026.101938 (PMC13331798; doi:10.1016/j.ssmph.2026.101938)
Supplement: Multimedia component 1 [file mmc1.docx]

**Appendix**

The appendix presents country-level statistics on trust in different types of healthcare providers in Table S1. Furthermore, Table S2 and Table S3 present sensitivity analyses for the main analysis in the main text (shown in Table 2). Table S2 demonstrates that the results of the main regression analysis remain stable when adding Covid-19-specific predictors on lockdown stringency and Covid-19 deaths. The results – the demonstrated relationships between GDP, personal income, and trust – remain stable as discussed in the main section of the paper. Notably, there are some significant relationships between the Covid-19 indicators and trust. Namely, countries with higher lockdown stringency tend to have higher levels of trust in healthcare providers, whereas higher death rates are associated with higher trust. These findings should be further investigated in future research, as they were not part of the main focus of this paper.

Table S3 uses a logistic regression model to replicate the main analysis (shown in Table 2) with an alternative variable coding (where 0 represents low trust and 1 represents some or a lot of trust). The findings and levels of statistical significance remain consistent with the results shown in the main text – demonstrating that the findings are not sensitive to the specific variable coding.

| **Table S1.** Descriptive statistics as a ranking, sorted by the mean county-level value of trust in doctors and nurses. Colour coding: darker colours (green or blue)  reflect higher correlation indices. | | | | | | | | | | | | | |
| --- | --- | --- | --- | --- | --- | --- | --- | --- | --- | --- | --- | --- | --- |
| **#** | **Country** | **Trust in doctors & nurses** | **Trust in hospitals & clinics** | **Trust in traditional healers** | **Correlate trust doctors and trust clinics** | **Correl. Doctors & trad. healers** | **#** | **Country** | **Trust in doctors & nurses** | **Trust in hospitals & clinics** | **Trust in trad. healers** | **Correl. Doctors & clinics** | **Correl. Doctors & trad. healers** |
| 1 | Belgium | **3.87** | 3.75 | 2.68 | .52 | .06 | 56 | Italy | **3.26** | 3.06 | 1.59 | .26 | .00 |
| 2 | Norway | **3.86** | 3.80 | 2.36 | .56 | -.02 | 57 | South Korea | **3.26** | 3.21 | 2.82 | .62 | .29 |
| 3 | Malta | **3.86** | 3.66 | 3.21 | .31 | .20 | 58 | Slovakia | **3.25** | 2.98 | 2.18 | .59 | .00 |
| 4 | Netherlands | **3.82** | 3.74 | 2.30 | .62 | -.04 | 59 | Cambodia | **3.24** | 3.21 | 2.49 | .43 | .06 |
| 5 | Finland | **3.80** | 3.80 | 2.09 | .58 | .05 | 60 | Colombia | **3.24** | 2.69 | 1.78 | .39 | .11 |
| 6 | Switzerland | **3.80** | 3.74 | 2.59 | .19 | .02 | 61 | Egypt | **3.23** | 2.59 | 2.77 | .40 | .16 |
| 7 | Australia | **3.78** | 3.66 | 2.58 | .52 | .07 | 62 | Nepal | **3.22** | 3.13 | 2.78 | .40 | .06 |
| 8 | France | **3.77** | 3.58 | 2.24 | .16 | -.06 | 63 | Hong Kong | **3.22** | 3.05 | 2.84 | .39 | .20 |
| 9 | Spain | **3.77** | 3.61 | 1.52 | .11 | -.11 | 64 | Senegal | **3.21** | 3.08 | 2.55 | .45 | .16 |
| 10 | New Zealand | **3.75** | 3.50 | 2.58 | .49 | -.05 | 65 | Lithuania | **3.20** | 3.01 | 1.99 | .62 | -.06 |
| 11 | Denmark | **3.74** | 3.65 | 2.55 | .62 | -.04 | 66 | Ethiopia | **3.20** | 2.98 | 2.33 | .41 | .07 |
| 12 | Austria | **3.74** | 3.69 | 2.63 | .15 | -.03 | 67 | El Salvador | **3.19** | 2.81 | 1.70 | .46 | .15 |
| 13 | Germany | **3.72** | 3.62 | 2.51 | .18 | .03 | 68 | Mongolia | **3.19** | 2.94 | 3.09 | .43 | .27 |
| 14 | Canada | **3.69** | 3.47 | 2.71 | .56 | .08 | 69 | Latvia | **3.19** | 2.98 | 1.78 | .61 | .08 |
| 15 | Sri Lanka | **3.65** | 3.41 | 3.29 | .41 | .08 | 70 | Poland | **3.16** | 2.78 | 2.26 | .59 | .01 |
| 16 | Ireland | **3.65** | 3.28 | 2.35 | .12 | -.09 | 71 | Kyrgyzstan | **3.14** | 2.96 | 2.37 | .54 | .21 |
| 17 | United States | **3.64** | 3.51 | 2.67 | .57 | .04 | 72 | Nicaragua | **3.13** | 2.98 | 1.49 | .52 | .09 |
| 18 | Czech Republic | **3.63** | 3.50 | 2.39 | .61 | -.01 | 73 | Bulgaria | **3.12** | 2.62 | 2.16 | .50 | .07 |
| 19 | Slovenia | **3.63** | 3.45 | 2.14 | .60 | .01 | 74 | Jordan | **3.12** | 2.87 | 2.83 | .58 | .09 |
| 20 | Sweden | **3.63** | 3.46 | 2.08 | .68 | .03 | 75 | Zambia | **3.11** | 2.79 | 1.49 | .34 | .00 |
| 21 | United Kingdom | **3.61** | 3.48 | 2.18 | .12 | -.03 | 76 | Serbia | **3.11** | 2.82 | 2.09 | .62 | .06 |
| 22 | Philippines | **3.60** | 3.28 | 2.34 | .40 | .14 | 77 | Lebanon | **3.09** | 2.47 | 2.33 | .57 | .22 |
| 23 | Argentina | **3.60** | 3.23 | 1.42 | .49 | -.01 | 78 | Kazakhstan | **3.09** | 3.00 | 2.02 | .54 | .15 |
| 24 | Israel | **3.60** | 3.44 | 2.64 | .60 | .07 | 79 | Uganda | **3.09** | 2.77 | 1.66 | .41 | .11 |
| 25 | Croatia | **3.58** | 3.30 | 1.99 | .49 | .03 | 80 | Ghana | **3.07** | 2.87 | 2.56 | .45 | .18 |
| 26 | Uruguay | **3.58** | 3.40 | 1.60 | .54 | .01 | 81 | Tunisia | **3.07** | 2.41 | 2.27 | .44 | .06 |
| 27 | Portugal | **3.54** | 3.28 | 2.77 | .51 | .19 | 82 | Romania | **3.07** | 2.48 | 2.12 | .54 | .15 |
| 28 | United Arab Emirates | **3.54** | 3.44 | 2.91 | .45 | .37 | 83 | Algeria | **3.06** | 2.80 | 2.96 | .60 | .03 |
| 29 | Malaysia | **3.49** | 3.37 | 2.01 | .38 | .09 | 84 | Morocco | **3.03** | 2.37 | 2.24 | .53 | .00 |
| 30 | Tanzania | **3.47** | 3.29 | 1.89 | .35 | .00 | 85 | Georgia | **3.03** | 2.84 | 2.53 | .58 | .21 |
| 31 | Thailand | **3.45** | 3.16 | 2.65 | .33 | .11 | 86 | Namibia | **3.03** | 2.74 | 1.72 | .32 | .03 |
| 32 | Turkey | **3.44** | 3.15 | 2.77 | .48 | .27 | 87 | Indonesia | **3.02** | 3.06 | 2.77 | .43 | .29 |
| 33 | Costa Rica | **3.43** | 3.27 | 1.58 | .56 | .09 | 88 | Paraguay | **3.02** | 2.68 | 2.33 | .44 | .10 |
| 34 | Uzbekistan | **3.42** | 3.26 | 2.23 | .61 | .24 | 89 | Bosnia Herzegovina | **3.01** | 2.60 | 1.96 | .58 | .11 |
| 35 | Taiwan | **3.42** | 3.44 | 2.69 | .53 | .16 | 90 | Burkina Faso | **3.00** | 2.70 | 2.64 | .42 | .26 |
| 36 | Laos | **3.41** | 3.30 | 2.94 | .44 | .17 | 91 | Montenegro | **2.98** | 2.77 | 2.04 | .61 | .06 |
| 37 | Estonia | **3.41** | 3.42 | 2.01 | .58 | .06 | 92 | Mauritius | **2.98** | 2.89 | 2.33 | .54 | .28 |
| 38 | Mexico | **3.41** | 2.89 | 1.73 | .42 | .01 | 93 | Kenya | **2.97** | 2.76 | 2.00 | .34 | .17 |
| 39 | India | **3.41** | 3.13 | 2.06 | .46 | .12 | 94 | Kosovo | **2.97** | 2.62 | 2.05 | .55 | .17 |
| 40 | Iran | **3.39** | 2.97 | 2.87 | .36 | .17 | 95 | Mali | **2.95** | 2.91 | 3.02 | .48 | .32 |
| 41 | South Africa | **3.39** | 2.96 | 2.39 | .31 | .16 | 96 | Peru | **2.92** | 2.46 | 1.35 | .40 | -.01 |
| 42 | Chile | **3.36** | 2.91 | 1.43 | .46 | .04 | 97 | Ecuador | **2.87** | 2.55 | 1.48 | .43 | .07 |
| 43 | Myanmar | **3.35** | 3.12 | 2.28 | .43 | .14 | 98 | North Macedonia | **2.86** | 2.43 | 2.24 | .54 | .24 |
| 44 | Hungary | **3.35** | 2.86 | 2.52 | .55 | .07 | 99 | Albania | **2.84** | 2.46 | 2.22 | .58 | .18 |
| 45 | Dominican Republic | **3.33** | 2.90 | 1.24 | .38 | .00 | 100 | Bolivia | **2.82** | 2.56 | 1.99 | .40 | .02 |
| 46 | Vietnam | **3.32** | 3.25 | 3.03 | .48 | .39 | 101 | Nigeria | **2.79** | 2.51 | 2.11 | .36 | .12 |
| 47 | Japan | **3.30** | 3.14 | 2.53 | .54 | .15 | 102 | Russia | **2.74** | 2.52 | 1.57 | .69 | .11 |
| 48 | Brazil | **3.30** | 2.81 | 1.86 | .44 | .10 | 103 | Moldova | **2.73** | 2.47 | 1.73 | .62 | .09 |
| 49 | Bahrain | **3.30** | n/a | 2.72 | n/a | .10 | 104 | Benin | **2.71** | 2.54 | 2.19 | .45 | .17 |
| 50 | Bangladesh | **3.30** | 3.09 | 1.48 | .48 | .01 | 105 | Ukraine | **2.71** | 2.40 | 1.64 | .62 | .11 |
| 51 | China | **3.29** | 3.14 | 3.30 | .47 | .42 | 106 | Ivory Coast | **2.69** | 2.50 | 2.47 | .45 | .13 |
| 52 | Saudi Arabia | **3.28** | 3.17 | 2.63 | .54 | .20 | 107 | Guinea | **2.69** | 2.54 | 2.28 | .38 | .20 |
| 53 | Zimbabwe | **3.27** | 2.90 | 1.95 | .51 | .06 | 108 | Iraq | **2.68** | 2.17 | 2.49 | .46 | .17 |
| 54 | Greece | **3.27** | 2.88 | 2.00 | .51 | .05 | 109 | Congo Brazzaville | **2.60** | 2.45 | 2.28 | .44 | .11 |
| 55 | Cyprus | **3.26** | 2.89 | 2.61 | .54 | .15 | 110 | Gabon | **2.59** | 2.35 | 2.03 | .42 | .12 |
|  |  |  |  |  |  |  | 111 | Cameroon | **2.48** | 2.33 | 2.02 | .39 | .17 |

|  | **Table S2.** Country-level statistics, Income groups are defined as follows: 4 = high-income countries, 3 = upper-middle-income countries, 2 = lower-middle-income countries, and 1 = low-income countries. | | | | | | | | | | |
| --- | --- | --- | --- | --- | --- | --- | --- | --- | --- | --- | --- |
|  | | **Sample size in survey dataset (n)** | **Income  group (year 2020)** | **GDP per capita (current US$)** | **Year, source** | **Health expen-diture (% of GDP)** | **Year, source** | **Out-of-pocket expenditure (% of health expen-diture)** | **Year,  source** | **Qaulity of care index** | **Corruption perception index** |
| Albania | | 1,000 | 3 | 5,246 | 2020, WB^1^ | 5.2 | 2019, WB^1^ | 44.6 | 2018, WB^1^ | 78.2 | 36 |
| United Arab Emirates | | 1,002 | 4 | 3,6285 | 2020, WB^1^ | 4.3 | 2019, WB^1^ | 12.5 | 2019, WB^1^ | 72.2 | 71 |
| Argentina | | 1,001 | 3 | 8,579 | 2020, WB^1^ | 9.5 | 2019, WB^1^ | 27.7 | 2019, WB^1^ | 68.4 | 42 |
| Australia | | 1,001 | 4 | 51,693 | 2020, WB^1^ | 9.9 | 2019, WB^1^ | 16.0 | 2019, WB^1^ | 89.8 | 77 |
| Austria | | 1,000 | 4 | 48,587 | 2020, WB^1^ | 10.4 | 2019, WB^1^ | 19.1 | 2019, WB^1^ | 88.2 | 76 |
| Belgium | | 1,001 | 4 | 45,159 | 2020, WB^1^ | 10.7 | 2019, WB^1^ | 18.2 | 2019, WB^1^ | 87.9 | 76 |
| Benin | | 1,007 | 2 | 1,291 | 2020, WB^1^ | 2.4 | 2019, WB^1^ | 47.0 | 2019, WB^1^ | 43 | 41 |
| Burkina Faso | | 1,002 | 1 | 858 | 2020, WB^1^ | 5.5 | 2019, WB^1^ | 34.7 | 2019, WB^1^ | 42.9 | 40 |
| Bangladesh | | 1,011 | 2 | 1,962 | 2020, WB^1^ | 2.5 | 2019, WB^1^ | 72.7 | 2019, WB^1^ | 51.7 | 26 |
| Bulgaria | | 1,007 | 3 | 10,079 | 2020, WB^1^ | 7.1 | 2019, WB^1^ | 39.0 | 2019, WB^1^ | 71.4 | 44 |
| Bahrain | | 1,005 | 4 | 20,410 | 2020, WB^1^ | 4.0 | 2019, WB^1^ | 29.7 | 2019, WB^1^ | 79 | 42 |
| Bosnia Herzegovina | | 1,002 | 3 | 6,080 | 2020, WB^1^ | 9.0 | 2019, WB^1^ | 29.4 | 2019, WB^1^ | 78.2 | 35 |
| Bolivia | | 1,002 | 2 | 3,133 | 2020, WB^1^ | 6.9 | 2019, WB^1^ | 23.9 | 2019, WB^1^ | 59.2 | 31 |
| Brazil | | 1,000 | 3 | 6,797 | 2020, WB^1^ | 9.6 | 2019, WB^1^ | 24.9 | 2019, WB^1^ | 64.9 | 38 |
| Canada | | 1,010 | 4 | 43,295 | 2020, WB^1^ | 10.8 | 2019, WB^1^ | 14.9 | 2019, WB^1^ | 87.6 | 77 |
| Switzerland | | 1,000 | 4 | 87,097 | 2020, WB^1^ | 11.3 | 2019, WB^1^ | 25.3 | 2019, WB^1^ | 91.8 | 85 |
| Chile | | 1,021 | 3 | 13,232 | 2020, WB^1^ | 9.3 | 2019, WB^1^ | 32.8 | 2019, WB^1^ | 76 | 67 |
| China | | 3,502 | 3 | 10,435 | 2020, WB^1^ | 5.4 | 2019, WB^1^ | 35.2 | 2019, WB^1^ | 74.2 | 42 |
| Cote d'Ivoire | | 1,005 | 2 | 2,326 | 2020, WB^1^ | 3.3 | 2019, WB^1^ | 37.3 | 2019, WB^1^ |  |  |
| Cameroon | | 1,006 | 2 | 1,537 | 2020, WB^1^ | 3.6 | 2019, WB^1^ | 72.5 | 2019, WB^1^ | 44.4 | 25 |
| Congo Brazzaville | | 1,009 | 2 | 1,846 | 2020, WB^1^ | 2.1 | 2019, WB^1^ | 45.9 | 2019, WB^1^ | 43.5 | 19 |
| Colombia | | 1,000 | 3 | 5,335 | 2020, WB^1^ | 7.7 | 2019, WB^1^ | 14.9 | 2019, WB^1^ | 67.8 | 39 |
| Costa Rica | | 1,001 | 3 | 12,141 | 2020, WB^1^ | 7.3 | 2019, WB^1^ | 22.3 | 2019, WB^1^ | 72.9 | 57 |
| Cyprus | | 1,012 | 4 | 27,528 | 2020, WB^1^ | 7.0 | 2019, WB^1^ | 30.6 | 2019, WB^1^ | 85.3 | 57 |
| Czech Republic | | 1,000 | 4 | 22,931 | 2020, WB^1^ | 7.8 | 2019, WB^1^ | 14.2 | 2019, WB^1^ | 84.8 | 54 |
| Germany | | 1,000 | 4 | 46,208 | 2020, WB^1^ | 11.7 | 2019, WB^1^ | 12.8 | 2019, WB^1^ | 86.4 | 80 |
| Denmark | | 1,000 | 4 | 61,063 | 2020, WB^1^ | 10.0 | 2019, WB^1^ | 14.2 | 2019, WB^1^ | 85.7 | 88 |
| Dominican Republic | | 1,000 | 3 | 7,268 | 2020, WB^1^ | 5.9 | 2019, WB^1^ | 42.9 | 2019, WB^1^ | 62.5 | 28 |
| Algeria | | 1,020 | 2 | 3,307 | 2020, WB^1^ | 6.2 | 2019, WB^1^ | 33.4 | 2019, WB^1^ | 63.7 | 36 |
| Ecuador | | 1,000 | 3 | 5,600 | 2020, WB^1^ | 7.8 | 2019, WB^1^ | 30.9 | 2019, WB^1^ | 61.2 | 39 |
| Egypt | | 1,004 | 2 | 3,569 | 2020, WB^1^ | 4.7 | 2019, WB^1^ | 62.7 | 2019, WB^1^ | 61 | 33 |
| Spain | | 1,000 | 4 | 27,063 | 2020, WB^1^ | 9.1 | 2019, WB^1^ | 21.8 | 2019, WB^1^ | 89.6 | 62 |
| Estonia | | 1,013 | 4 | 23,027 | 2020, WB^1^ | 6.7 | 2019, WB^1^ | 24.0 | 2019, WB^1^ | 81.4 | 75 |
| Ethiopia | | 1,003 | 1 | 936 | 2020, WB^1^ | 3.2 | 2019, WB^1^ | 37.9 | 2019, WB^1^ | 44.2 | 38 |
| Finland | | 1,000 | 4 | 48,745 | 2020, WB^1^ | 9.2 | 2019, WB^1^ | 17.4 | 2019, WB^1^ | 89.6 | 85 |
| France | | 1,000 | 4 | 39,030 | 2020, WB^1^ | 11.1 | 2019, WB^1^ | 9.3 | 2019, WB^1^ | 87.9 | 69 |
| Gabon | | 1,005 | 3 | 6,882 | 2020, WB^1^ | 2.8 | 2019, WB^1^ | 23.1 | 2019, WB^1^ | 51.4 | 30 |
| United Kingdom | | 1,000 | 4 | 41,059 | 2020, WB^1^ | 10.2 | 2019, WB^1^ | 17.1 | 2019, WB^1^ | 84.6 | 77 |
| Georgia | | 1,000 | 3 | 4,267 | 2020, WB^1^ | 6.7 | 2019, WB^1^ | 46.8 | 2019, WB^1^ | 62.1 | 56 |
| Ghana | | 1,000 | 2 | 2,206 | 2020, WB^1^ | 3.4 | 2019, WB^1^ | 36.2 | 2019, WB^1^ | 49.7 | 43 |
| Guinea | | 1,009 | 1 | 1,194 | 2020, WB^1^ | 4.0 | 2019, WB^1^ | 59.2 | 2019, WB^1^ | 38.6 | 28 |
| Greece | | 1,006 | 4 | 17,623 | 2020, WB^1^ | 7.8 | 2019, WB^1^ | 35.2 | 2019, WB^1^ | 87 | 50 |
| Hong Kong | | 1,004 | 4 | 46,324 | 2020, WB^1^ | 6.5 | 2019, ^^[[1]](#footnote-2)^^ | 30.0 | 2019, WB^^[[2]](#footnote-3)^^ |  |  |
| Croatia | | 1,000 | 4 | 14,134 | 2020, WB^1^ | 7.0 | 2019, WB^1^ | 11.5 | 2019, WB^1^ | 81.6 | 47 |
| Hungary | | 1,000 | 4 | 15,981 | 2020, WB^1^ | 6.4 | 2019, WB^1^ | 28.2 | 2019, WB^1^ | 79.6 | 44 |
| Indonesia | | 1,023 | 3 | 3,870 | 2020, WB^1^ | 2.9 | 2019, WB^1^ | 34.8 | 2019, WB^1^ | 49.2 | 37 |
| India | | 3,045 | 2 | 1,928 | 2020, WB^1^ | 3.0 | 2019, WB^1^ | 54.8 | 2019, WB^1^ | 44.8 | 40 |
| Ireland | | 1,000 | 4 | 85,268 | 2020, WB^1^ | 6.7 | 2019, WB^1^ | 11.7 | 2019, WB^1^ | 88.4 | 72 |
| Iran | | 1,007 | 3 | 2,422 | 2020, WB^1^ | 6.7 | 2019, WB^1^ | 39.5 | 2019, WB^1^ | 71.1 | 25 |
| Iraq | | 1,009 | 3 | 4,146 | 2020, WB^1^ | 4.5 | 2019, WB^1^ | 50.1 | 2019, WB^1^ | 60.1 | 21 |
| Israel | | 1,063 | 4 | 44,169 | 2020, WB^1^ | 7.5 | 2019, WB^1^ | 21.0 | 2019, WB^1^ | 85.5 | 60 |
| Italy | | 1,000 | 4 | 31,714 | 2020, WB^1^ | 8.7 | 2019, WB^1^ | 23.3 | 2019, WB^1^ | 88.7 | 53 |
| Jordan | | 1,005 | 3 | 4,283 | 2020, WB^1^ | 7.6 | 2019, WB^1^ | 30.3 | 2019, WB^1^ | 76.5 | 49 |
| Japan | | 1,012 | 4 | 40,193 | 2020, WB^1^ | 10.7 | 2019, WB^1^ | 12.9 | 2019, WB^1^ | 89 | 74 |
| Kazakhstan | | 1,000 | 3 | 9,122 | 2020, WB^1^ | 2.8 | 2019, WB^1^ | 33.9 | 2019, WB^1^ | 61.1 | 38 |
| Kenya | | 1,002 | 2 | 1,879 | 2020, WB^1^ | 4.6 | 2019, WB^1^ | 24.3 | 2019, WB^1^ | 48.7 | 31 |
| Kyrgyzstan | | 1,000 | 2 | 1,174 | 2020, WB^1^ | 4.5 | 2019, WB^1^ | 46.2 | 2019, WB^1^ | 60.4 | 31 |
| Cambodia | | 1,000 | 2 | 1,544 | 2020, WB^1^ | 7.0 | 2019, WB^1^ | 64.4 | 2019, WB^1^ | 50.7 | 21 |
| South Korea | | 1,009 | 4 | 31,631 | 2020, WB^1^ | 8.2 | 2019, WB^1^ | 30.2 | 2019, WB^1^ | 85.8 | 61 |
| Laos | | 1,000 | 2 | 2,630 | 2020, WB^1^ | 2.6 | 2019, WB^1^ | 41.8 | 2019, WB^1^ | 44.9 | 29 |
| Lebanon | | 1,035 | 3 | 4,650 | 2020, WB^1^ | 8.6 | 2019, WB^1^ | 33.5 | 2019, WB^1^ | 80 | 25 |
| Sri Lanka | | 1,011 | 2 | 3,681 | 2020, WB^1^ | 4.1 | 2019, WB^1^ | 45.6 | 2019, WB^1^ | 72.8 | 38 |
| Lithuania | | 1,001 | 4 | 20,234 | 2020, WB^1^ | 7.0 | 2019, WB^1^ | 32.3 | 2019, WB^1^ | 76.6 | 60 |
| Latvia | | 1,005 | 4 | 17,726 | 2020, WB^1^ | 6.6 | 2019, WB^1^ | 35.7 | 2019, WB^1^ | 77.7 | 57 |
| Morocco | | 1,012 | 2 | 3,059 | 2020, WB^1^ | 5.3 | 2019, WB^1^ | 46.8 | 2019, WB^1^ | 61.3 | 40 |
| Moldova | | 1,005 | 2 | 4,547 | 2020, WB^1^ | 6.4 | 2019, WB^1^ | 35.7 | 2019, WB^1^ | 73.1 | 34 |
| Mexico | | 1,000 | 3 | 8,329 | 2020, WB^1^ | 5.4 | 2019, WB^1^ | 42.1 | 2019, WB^1^ | 62.6 | 31 |
| North Macedonia | | 1,019 | 3 | 5,917 | 2020, WB^1^ | 7.3 | 2019, WB^1^ | 40.4 | 2019, WB^1^ | 76 | 35 |
| Mali | | 1,002 | 1 | 862 | 2020, WB^1^ | 3.9 | 2019, WB^1^ | 31.4 | 2019, WB^1^ | 45.6 | 30 |
| Malta | | 1,002 | 4 | 27,885 | 2020, WB^1^ | 8.2 | 2019, WB^1^ | 34.6 | 2019, WB^1^ | 85.1 | 53 |
| Myanmar | | 1,000 | 2 | 1,468 | 2020, WB^1^ | 4.7 | 2019, WB^1^ | 76.0 | 2019, WB^1^ | 48.4 | 28 |
| Montenegro | | 1,027 | 3 | 7,677 | 2020, WB^1^ | 8.3 | 2019, WB^1^ | 38.6 | 2019, WB^1^ | 80.7 | 45 |
| Mongolia | | 1,000 | 2 | 4,061 | 2020, WB^1^ | 3.8 | 2019, WB^1^ | 34.8 | 2019, WB^1^ | 58.5 | 35 |
| Mauritius | | 1,000 | 4 | 8,628 | 2020, WB^1^ | 6.2 | 2019, WB^1^ | 45.7 | 2019, WB^1^ | 65.7 | 53 |
| Malaysia | | 1,004 | 3 | 10,412 | 2020, WB^1^ | 3.8 | 2019, WB^1^ | 34.6 | 2019, WB^1^ | 66.6 | 51 |
| Namibia | | 1,007 | 3 | 4,179 | 2020, WB^1^ | 8.5 | 2019, WB^1^ | 8.2 | 2019, WB^1^ | 53.7 | 51 |
| Nigeria | | 1,002 | 2 | 2,097 | 2020, WB^1^ | 3.0 | 2019, WB^1^ | 70.5 | 2019, WB^1^ | 51.3 | 25 |
| Nicaragua | | 1,000 | 2 | 1,905 | 2020, WB^1^ | 8.4 | 2019, WB^1^ | 34.4 | 2019, WB^1^ | 64.3 | 22 |
| Netherlands | | 1,000 | 4 | 52,397 | 2020, WB^1^ | 10.1 | 2019, WB^1^ | 10.6 | 2019, WB^1^ | 89.5 | 82 |
| Norway | | 1,000 | 4 | 67,330 | 2020, WB^1^ | 10.5 | 2019, WB^1^ | 13.9 | 2019, WB^1^ | 90.5 | 84 |
| Nepal | | 1,000 | 2 | 1,155 | 2020, WB^1^ | 4.4 | 2019, WB^1^ | 57.9 | 2019, WB^1^ | 50.8 | 33 |
| New Zealand | | 1,000 | 4 | 41,441 | 2020, WB^1^ | 9.7 | 2019, WB^1^ | 12.2 | 2019, WB^1^ | 86.2 | 88 |
| Peru | | 1,001 | 3 | 6,127 | 2020, WB^1^ | 5.2 | 2019, WB^1^ | 28.1 | 2019, WB^1^ | 69.6 | 38 |
| Philippines | | 1,000 | 2 | 3,299 | 2020, WB^1^ | 4.1 | 2019, WB^1^ | 48.6 | 2019, WB^1^ | 52 | 34 |
| Poland | | 1,002 | 4 | 15,721 | 2020, WB^1^ | 6.4 | 2019, WB^1^ | 20.4 | 2019, WB^1^ | 79.6 | 56 |
| Portugal | | 1,004 | 4 | 22,176 | 2020, WB^1^ | 9.5 | 2019, WB^1^ | 30.5 | 2019, WB^1^ | 84.5 | 61 |
| Paraguay | | 1,000 | 3 | 5,001 | 2020, WB^1^ | 7.2 | 2019, WB^1^ | 41.6 | 2019, WB^1^ | 60.4 | 28 |
| Romania | | 1,006 | 4 | 12,896 | 2020, WB^1^ | 5.7 | 2019, WB^1^ | 18.9 | 2019, WB^1^ | 74.4 | 44 |
| Russia | | 2,002 | 3 | 10,127 | 2020, WB^1^ | 5.6 | 2019, WB^1^ | 36.6 | 2019, WB^1^ | 71.7 | 30 |
| Saudi Arabia | | 1,013 | 4 | 20,110 | 2020, WB^1^ | 5.7 | 2019, WB^1^ | 16.5 | 2019, WB^1^ | 79.4 | 53 |
| Senegal | | 1,025 | 2 | 1,472 | 2020, WB^1^ | 4.1 | 2019, WB^1^ | 51.0 | 2019, WB^1^ | 44.4 | 45 |
| El Salvador | | 1,000 | 2 | 3,799 | 2020, WB^1^ | 7.2 | 2019, WB^1^ | 28.4 | 2019, WB^1^ | 64.4 | 36 |
| Serbia | | 1,000 | 3 | 7,721 | 2020, WB^1^ | 8.7 | 2019, WB^1^ | 37.0 | 2019, WB^1^ | 75.4 | 38 |
| Slovakia | | 1,004 | 4 | 19,267 | 2020, WB^1^ | 7.0 | 2019, WB^1^ | 19.2 | 2019, WB^1^ | 78.6 | 49 |
| Slovenia | | 1,001 | 4 | 25,517 | 2020, WB^1^ | 8.5 | 2019, WB^1^ | 11.7 | 2019, WB^1^ | 87.4 | 60 |
| Sweden | | 1,000 | 4 | 52,274 | 2020, WB^1^ | 10.9 | 2019, WB^1^ | 13.9 | 2019, WB^1^ | 90.5 | 85 |
| Thailand | | 1,000 | 3 | 7,187 | 2020, WB^1^ | 3.8 | 2019, WB^1^ | 8.7 | 2019, WB^1^ | 70.8 | 36 |
| Tunisia | | 1,006 | 2 | 3,522 | 2020, WB^1^ | 7.0 | 2019, WB^1^ | 37.9 | 2019, WB^1^ | 70.1 | 44 |
| Turkey | | 1,000 | 3 | 8,536 | 2020, WB^1^ | 4.3 | 2019, WB^1^ | 16.9 | 2019, WB^1^ | 76.2 | 40 |
| Tanzania | | 1,000 | 2 | 1,076 | 2020, WB^1^ | 3.8 | 2019, WB^1^ | 22.2 | 2019, WB^1^ | 49.9 | 38 |
| Uganda | | 1,027 | 1 | 822 | 2020, WB^1^ | 3.8 | 2019, WB^1^ | 38.3 | 2019, WB^1^ | 42.9 | 27 |
| Ukraine | | 1,000 | 2 | 3,725 | 2020, WB^1^ | 7.1 | 2019, WB^1^ | 51.1 | 2019, WB^1^ | 72.7 | 33 |
| Uruguay | | 1,003 | 4 | 15,438 | 2020, WB^1^ | 9.3 | 2019, WB^1^ | 15.5 | 2019, WB^1^ | 72 | 71 |
| United States | | 1,001 | 4 | 63,593 | 2020, WB^1^ | 16.8 | 2019, WB^1^ | 11.3 | 2019, WB^1^ | 81.3 | 67 |
| Uzbekistan | | 1,000 | 2 | 1,751 | 2020, WB^1^ | 5.6 | 2019, WB^1^ | 57.7 | 2019, WB^1^ | 62.3 | 26 |
| Vietnam | | 1,000 | 2 | 2,786 | 2020, WB^1^ | 5.2 | 2019, WB^1^ | 43.0 | 2019, WB^1^ | 66.3 | 36 |
| Kosovo | | 1,004 | 3 | 4,347 | 2020, WB^1^ | 4.5 | 2017, ^^[[3]](#footnote-4)^^ | 33.3 | 2014, ^^[[4]](#footnote-5)^^ |  |  |
| South Africa | | 1,004 | 3 | 5,656 | 2020, WB^1^ | 9.1 | 2019, WB^1^ | 5.7 | 2019, WB^1^ | 52 | 44 |
| Zambia | | 1,005 | 2 | 985 | 2020, WB^1^ | 5.3 | 2019, WB^1^ | 10.2 | 2019, WB^1^ | 41.6 | 33 |
| Zimbabwe | | 1,002 | 2 | 1,215 | 2020, WB^1^ | 7.7 | 2019, WB^1^ | 24.4 | 2019, WB^1^ | 48.7 | 24 |
| Taiwan | | 1,000 | 4 | 28,358 | 2020, ^^[[5]](#footnote-6)^^ | 6.1 | 2017, ^^[[6]](#footnote-7)^^ | 34.0 | 2016, ^^[[7]](#footnote-8)^^ | 77.6 | 65 |

| **Table S3.** Multi-level regression model with Covid controls. | | | | | | | | |
| --- | --- | --- | --- | --- | --- | --- | --- | --- |
|  | **Outcome: Trust in health professionals** | | | | **Outcome: Trust in traditional health practitioners** | | | |
| ***Individual-level factors*** | **M1 (individual level)** | **M2 (income *GDP)** | **M3 (quadratic model)** | **M4 (country-level)** | **M5  (individual level)** | **M6 (Income* GDP)** | **M7 (quadratic model)** | **M8 (country-level)** |
| Age | **.02*** [.01 – .02] | x | x | x | **-.05*** [-.06 – .05] | x | x | x |
| Gender (being male) | **-.02**** [-.03 – -.01] | x | x | x | **-.03**** [-.04 – -.02] | x | x | x |
| Primary school or less (ref= secondary school) | -.01 [-.02 – .00] | x | x | x | **.07**** [.06 – .09] | x | x | x |
| University education | .**04**** [.03 – .05] | x | x | x | **-.08**** [-.10 – -.06] | x | x | x |
| Household income quintile 3 (ref= quintile 1) | **.03**** [.02 – .04] | **-.19**** [-.30 – -.09] | **.04**** [.03 – .06] | x | **-.04**** [-.06 – -.02] | -.11 [-.25 – .02] | **-.04**** [-.05 – -.02] | x |
| Household income quintile 5 (ref= quintile 1) | **.04**** [.03 – .06] | **-.28**** [-.39 – -.18] | **.05**** [.04 – .07] | x | **-.08*** [-.09 – -.06] | .01 [-.12 – .15] | **-.07**** [-.09 – -.05] | x |
| Quintile 3 * Log(GDP) |  | **.03**** [.02 – .04] |  |  |  | -.01 [-.01 – .02] |  |  |
| Quintile 5 * Log(GDP) |  | **.04**** [.03 – .05] |  |  |  | -.01 [-.02 – .01] |  |  |
| Trust in neighbours | **.20**** [.19 – .20] |  |  | x | **.14**** [.13 – .14] |  |  | x |
| ***Country-level factors*** |  |  |  |  |  |  |  |  |
| Log(GDP) |  | **.13**** [.09 – .18] | **-.97**** [-1.51 – -.44] | -.04 [-.04 - .14] |  | **.13** [.05 – .21]** | -.39 [-1.41 – .63] | -.01 [-.21 - .20] |
| Log(GDP)^2 |  |  | **.06**** [.03 – .09] |  |  |  | 0.03 [-.03 – .08] |  |
| Corruption index |  |  |  | **.14**** [.06 – .22] |  |  |  | .04 [-.13 – .21] |
| Health expenditure as % of GDP |  |  |  | .04 [-.02 - .11] |  |  |  | -.02 [-.16– .11] |
| % Out of Pocket healthcare expenditure |  |  |  | -.04 [-.10 - .01] |  |  |  | .05 [-.06 – .16] |
| Quality of care index |  |  |  | .03 [-.06 – .13] |  |  |  | .18 [-.02 – .38] |
| ***Covid-19 indicators*** |  |  |  |  |  |  |  |  |
| Lockdown stringency |  | **.07**** [.02 – .12] | **.08**** [.03 – .12] | **.08**** [.03 – .12] |  | **.10**** [.01 – .19] | **.10**** [.01 – .19] | .09 [.01 – .19] |
| Covid-19 deaths |  | **-.03**** [-.06 – -.01] | **-.02** [-.04 – -.01] | **-.03*** [-.05 – -.00] |  | **-.11**** [-.16 – -.07] | **-.11**** [-.15 – -.06] | **-.12**** [-.17 – -.06] |
| Marginal R^2^ | 0.053 | 0.057 | 0.069 | 0.121 | 0.018 | 0.006 | 0.015 | 0.033 |
| Conditional R^2^ | 0.167 | 0.152 | 0.150 | 0.190 | 0.207 | 0.201 | 0.202 | 0.214 |
| *Notes:* "x” indicates that the variable was included as control variable in the model; 111 countries included. Several country-level factors (corruption index, healthcare expenditure, out of pocket expenditure and quality of care, lockdown stringency) were standardised so that a change of 1 level = 1 SD, Covid-19-related deaths per 1000,000 inhabitants (until 31st Jan 2021); ** p <0.01, * p <0.05. | | | | | | | | |

| **Table S4.** Logistic regression model on the probability of high trust in healthcare providers | | | | | | | | |
| --- | --- | --- | --- | --- | --- | --- | --- | --- |
|  | **Outcome: Trust in health professionals** | | | | **Outcome: Trust in traditional health practitioners** | | | |
| ***Individual-level factors*** | **M1 (individual level)** | **M2 (income *GDP)** | **M3 (quadratic model)** | **M4 (country-level)** | **M5  (individual level)** | **M6 (Income* GDP)** | **M7 (quadratic model)** | **M8 (country-level)** |
| Age | **1.05**** [1.02 – 1.07] | x | x | x | **0.90**** [0.89 – 0.92] | x | x | x |
| Gender (being male) | **0.86**** [0.83 – 0.90] | x | x | x | **0.94**** [0.91 – 0.97] | x | x | x |
| Primary school or less (ref= secondary school) | **0.82**** [0.78 – 0.86] | x | x | x | **1.11**** [1.07 – 1.15] | x | x | x |
| University education | **1.30**** [1.21 – 1.39] | x | x | x | **0.82**** [0.79 – 0.86] | x | x | x |
| Household income quintile 3 (ref= quintile 1) | **1.14**** [1.08 – 1.21] | **0.44**** [0.28 – 0.68] | **1.17**** [1.10 – 1.23] | x | **0.92**** [0.88 – 0.97] | 1.00 [0.74 – 1.35] | **0.94*** [0.90 – 0.98] | x |
| Household income quintile 5 (ref= quintile 1) | **1.21**** [1.13 – 1.28] | **0.25**** [0.16 – 0.40] | **1.22**** [1.15 – 1.29] | x | **0.87**** [0.83 – 0.92] | 1.19** [0.87 – 1.63] | 0.88 [0.85 – 0.92] | x |
| Quintile 3 * Log(GDP) |  | **1.12**** [1.07 – 1.18] |  |  |  | 0.99 [0.96 – 1.03] |  |  |
| Quintile 5 * Log(GDP) |  | **1.20**** [1.14 – 1.27] |  |  |  | 0.97 [0.93 – 1.00] |  |  |
| Trust in neighbours | **1.66**** [1.63 – 1.70] |  |  | x | **1.32**** [1.30 – 1.34] |  |  | x |
| ***Country-level factors*** |  |  |  |  |  |  |  |  |
| Log(GDP) |  | **1.40**** [1.23 – 1.59] | 0.03 [0.00 – 2.75] | 0.90 [0.63 – 1.28] |  | 1.11 [0.96 – 1.28] | 0.16 [0.02 – 1.15] | 0.87 [0.61 – 1.24] |
| Log(GDP)^2 |  |  | 1.25 [0.97 – 1.60] |  |  |  | 1.11 [1.00 – 1.25] |  |
| Corruption index |  |  |  | **1.66**** [1.24 – 2.23] |  |  |  | 1.33 [0.96 – 1.86] |
| Health expenditure as % of GDP |  |  |  | 1.04 [0.83 – 1.30] |  |  |  | **0.77*** [0.60 – 0.99] |
| % Out of Pocket healthcare expenditure |  |  |  | 1.01 [0.83 – 1.22] |  |  |  | 1.12 [0.90 – 1.40] |
| Quality of care index |  |  |  | 1.17 [0.78 – 1.75] |  |  |  | 1.30 [0.88 – 1.92] |
| Marginal R^2^ | 0.062 | 0.103 | 0.121 | 0.171 | 0.019 | 0.005 | 0.012 | 0.034 |
| Conditional R^2^ | 0.254 | 0.258 | 0.249 | 0.284 | 0.220 | 0.212 | 0.212 | 0.220 |
| *Notes:* "x” indicates that the variable was included as control variable in the model; 111 countries included. Several country-level factors (corruption index, healthcare expenditure, out of pocket expenditure and quality of care, lockdown stringency) were standardised so that a change of 1 level = 1 SD. ; ** p <0.01, * p <0.05. | | | | | | | | |

1. https://www.healthbureau.gov.hk/statistics/download/dha/en/table1_1920.pdf [↑](#footnote-ref-2)
2. [https://www.healthbureau.gov.hk/statistics/en/dha/dha_summary_report.htm#:~:text=Analysed%20by%20financing%20scheme%2C%2053](https://www.healthbureau.gov.hk/statistics/en/dha/dha_summary_report.htm%23:~:text=Analysed%20by%20financing%20scheme%2C%2053) [↑](#footnote-ref-3)
3. Kosovo National Health Accounts Report for 2017 <https://msh.rks-gov.net/wp-content/uploads/2019/10/Raporti-p%C3%ABr-NHA-ENG.pdf> [↑](#footnote-ref-4)
4. Arenliu Qosaj, F., Froeschl, G., Berisha, M., Bellaqa, B., & Holle, R. (2018). Catastrophic expenditures and impoverishment due to out-of-pocket health payments in Kosovo. *Cost effectiveness and resource allocation*, *16*(1), 1-12. [↑](#footnote-ref-5)
5. <https://www.statista.com/statistics/727592/gross-domestic-product-gdp-per-capita-in-taiwan/> [↑](#footnote-ref-6)
6. <https://www.healthaffairs.org/do/10.1377/forefront.20190206.305164/#:~:text=National%20health%20expenditure%20(NHE)%20in,the%20average%20for%20OECD%20countries.> [↑](#footnote-ref-7)
7. https://www.commonwealthfund.org/international-health-policy-center/countries/taiwan [↑](#footnote-ref-8)
